# Extra Tree Classifier Predicts an Interactome Hub Gene as HSPB1 in Oral Cancer: A Bioinformatics Analysis

**DOI:** 10.7759/cureus.59863

**Published:** 2024-05-08

**Authors:** Deepavalli Arumuganainar

**Affiliations:** 1 Department of Periodontics, Saveetha Dental College and Hospitals, Saveetha Institute of Medical and Technical Sciences, Saveetha University, Chennai, IND

**Keywords:** interactome analysis, hspb1, hub genes, oral cancer, interactome

## Abstract

Introduction

Oral cancer is a significant global health issue that is mainly caused by factors, such as smoking, alcohol consumption, poor oral hygiene, age, and the human papillomavirus. Unfortunately, delayed diagnosis contributes to high rates of illness and mortality. However, saliva shows promise as a potential source for early detection, prognosis, and treatment. By analyzing the proteins and their interactions in saliva, we can gain insights that can assist in early detection and prediction. In this study, we aim to identify and predict the key genes, known as hub genes, in the salivary transcriptomics data of oral cancer patients and healthy individuals.

Methods

The data used for the analysis were obtained from salivaryproteome.org (https://salivaryproteome.org/) . The retrieved data consisted of individuals with oral cancer who were assigned unique identification numbers (IDs) 1025, 1030, 1027, and 1029, while the healthy individuals were assigned IDs 4256, 4257, 4255, and 4258, respectively. Differential gene expression analysis was used to identify genes that showed significant differences between the two groups. Uniformity and clustering were assessed through heatmaps and principal component analysis. Protein-protein interactions were investigated using the STRING database and Cytoscape. In addition, machine learning algorithms were employed to identify key genes involved in the interatomic interactions by analyzing transcriptomics data generated from the differential gene expression analysis.

Results

The accuracy and class accuracy of the extra tree classifier showed 98% and 97% in predicting interactomic hub genes, and HSPB1 was identified as a hub gene using Cytohubba from Cytoscape.

Conclusion

The predictive extra tree classifier, with its high accuracy in analysing interactomic hub genes in oral cancer, can potentially improve diagnosis and treatment strategies.

## Introduction

Oral cancer, a type of head and neck cancer, is a perilous and potentially life-threatening condition affecting the oral cavity, including the lips, tongue, gingiva, cheeks, and roof of the mouth [1,2]. It can be caused by smoking, alcohol consumption, a family history of cancer, and exposure to chemicals. Symptoms include persistent mouth sores, swelling, difficulty chewing, changes in voice or speech, and unexplained weight loss. Treatment options include surgical therapy, chemotherapy, radiation therapy, targeted therapy, or a combination of the above. Prevention involves avoiding risk factors, regular dental check-ups, and the HPV vaccine [3]. Advancements in medical technology and awareness have improved diagnosis and treatment outcomes. In addition, craniofacial tumors are known to present diverse clinical behaviors and histopathological presentations. A previous clinicopathological study of craniofacial tumor management was conducted on 319 patients and identified that the unique nature of cystic and neoplastic pathosis in the craniofacial region was due to aesthetic and functional derangements, with variations in prevalence due to occupational, sociocultural, and climatic factors [4].

The oral cancer interactome hub refers to a network of interactions between proteins involved in the development and progression of oral cancer. An interactome is a comprehensive map of interactions between proteins within a cell or biological system. The oral cancer interactome hub [5-7] is a research concept that aims to understand molecular pathways and protein-protein interactions in oral cancer. It uses experimental techniques and computational methods to map interactions, identify biomarkers, and develop new treatment strategies [8,9]. This knowledge can lead to targeted therapies that improve treatment outcomes for patients.

A previous study proposes a protein-protein interaction network technique to analyze genes involved in oral cancer disorders, revealing a network with 208 nodes and 1572 edges. TP53, a key gene [10,11], is identified as a key player in the oral cancer network, enhancing disease understanding and treatment. A study analyzing saliva proteomics data for oral cancer identified 74 candidate genes or proteins. The analysis identified nine hub-bottleneck proteins, including kininogen-1, angiotensinogen, annexin A1, IL-8, IgG heavy chains, CRP, collagen alpha-1, and fibronectin, as potential biomarkers for diagnosis and treatment. A study uses high-throughput transcriptomics methods and machine learning to identify disease biomarkers for MetS. The integrated approach uses WGCNA, LASSO regression, and RF algorithms for feature selection. A logistic regression model and web nomogram calculator are created for MetS risk assessment, aiming to develop reliable biomarkers [12]. Machine learning is used to predict and analyze complex interactions within biological networks, particularly in interactome hub genes. These genes play crucial roles in cellular processes and are often linked to disease mechanisms. Techniques like clustering, classification, and network analysis help identify key hub genes, predict gene function, discover disease biomarkers, and understand genetic perturbations.

Oral cancer results in high mortality rates and limited treatment options. This study aimed to identify interactomic hub genes associated with oral cancer and propose a predictive model for their identification by using differential expression analysis to identify genes significantly dysregulated in oral cancer and extract protein-protein interaction data from publicly available databases. Using functional enrichment analysis, the study will validate the identified hub genes' significance and functional relevance. A predictive model will be developed to classify oral cancer patients into subtypes or predict disease outcomes. Hence, our study is based on machine learning prediction of interactome hub genes in oral cancer.

## Materials and methods

The data used for the analysis were obtained from salivaryproteome.org. The retrieved data consisted of individuals with oral cancer who were assigned unique identification numbers (IDs) 1025, 1030, 1027, and 1029, while the healthy individuals were assigned IDs 4256, 4257, 4255, and 4258, respectively. 

This study acknowledges the importance of integrated bioinformatics analysis in identifying hub genes using network analysis. This approach allows us to leverage the power of AI algorithms to predict hub genes, leading to the discovery of potential biomarkers and drug targets in oral cancer. The study aims to contribute to the field by shedding light on the intricacies of oral cancer at a molecular level. By utilizing network analysis and applying advanced AI algorithms, the key genes that play a crucial role in the development and progression of oral cancer may be uncovered. Thus, the findings may significantly enhance our understanding of oral cancer pathogenesis and potentially pave the way for developing novel therapeutic strategies.

Differentially expressed gene (DEG) analysis was performed to identify genes that exhibited significant changes in expression between the oral squamous cell carcinoma (OSCC) group and the healthy group. A heatmap was generated using the ggplot2 package to assess the uniformity of the samples. In addition, clustering analysis was performed using principal component analysis (PCA) to explore the overall gene expression patterns between the OSCC and healthy groups. DEG analysis was carried out using the limma package in R (version 4.1.0). Linear models were constructed to identify DEGs based on their expression levels. Genes with an absolute log2 (fold change) greater than 1.0 and an adjusted p-value of <0.05 were considered significant DEGs.

A Venn diagram was generated to identify critical genes common to the cancer and healthy groups and display the overlap between the samples. This allowed for the identification of genes that may have a potential role in the development or progression of oral cancer. To investigate protein-protein interactions (PPIs), the STRING database was utilized to construct a PPI network. This network provides insights into the interactions and functions of proteins associated with oral cancer. Subsequently, the PPI network was visualized using Cytoscape, a powerful network visualization and analysis tool. Furthermore, the cytoHubba plugin for Cytoscape was employed to identify the top 10 hub genes using the maximal clique centrality algorithm.

Extra tree classifier

The extra tree classifier is an ensemble-based machine learning algorithm, a variant of the decision tree algorithm. It consists of many individual decision trees trained independently and in parallel. Each tree is trained with random feature selection, threshold selection, and majority voting. After training, each tree independently predicts the target class based on input features. The class label with the most votes is selected as the predicted class label. This architecture introduces randomness to reduce overfitting and increase the diversity of individual decision trees, leading to improved model accuracy and generalization.

## Results

**Figure 1:**
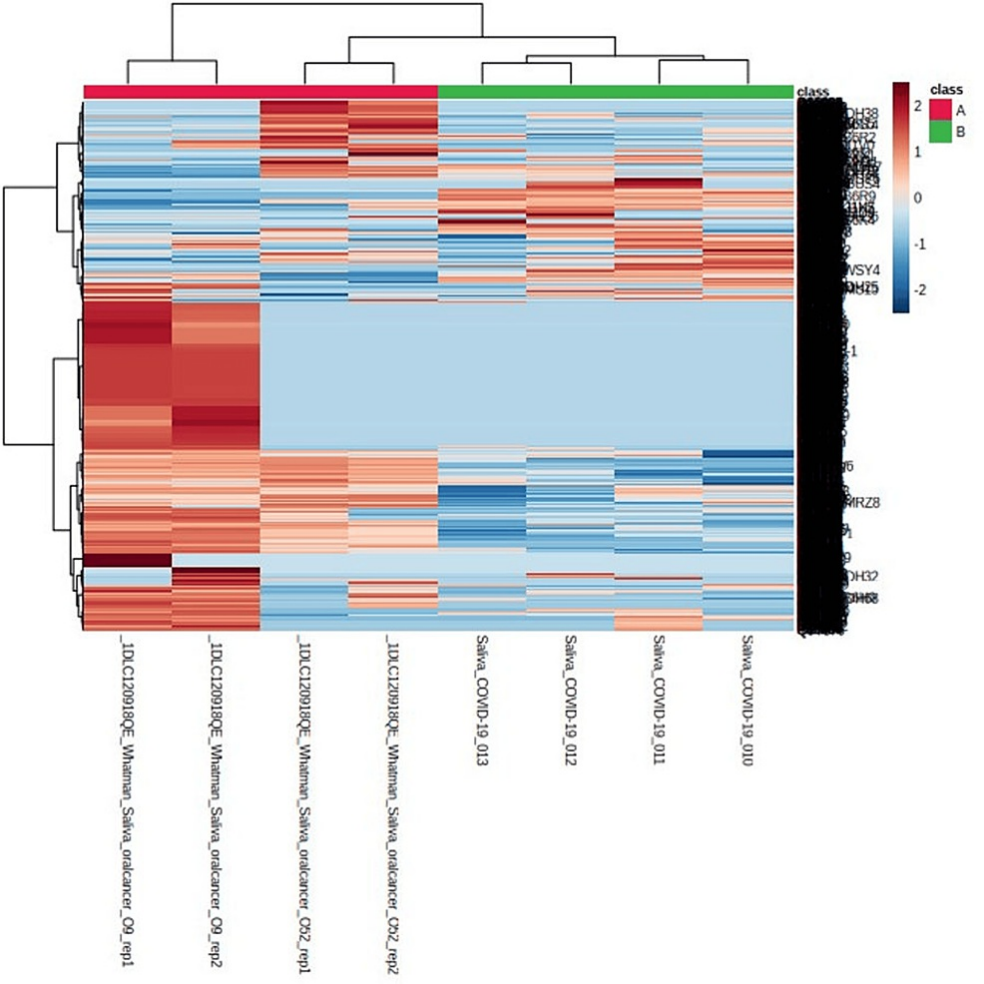
Heat map of cancer and normal sample distribution

The results show a heat map of cancer and normal sample distribution. The color scale represents the gene expression levels, with blue indicating low expression, white indicating medium expression, and red indicating high expression. The "annotation_col" argument assigns a different color or annotation to the cancer and normal samples to distinguish them in the heatmap (Figure 1).

**Figure 2:**
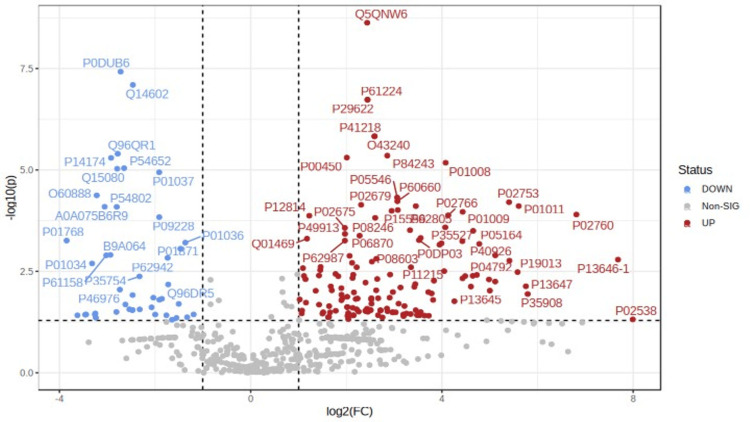
Volcano plot providing a snapshot of changes in gene expression levels and their statistical significance

The volcano plot provides a snapshot of changes in gene expression levels and their statistical significance. The plot uses color to differentiate genes based on their regulation status, with upregulated genes marked in red and downregulated genes in blue. The position and color of each gene point provide insights into its significance and direction of regulation. The interpretation of a volcano plot should consider the significance threshold, fold change cutoff values, and potential sources of noise and biases in the data (Figure 2).

**Figure 3:**
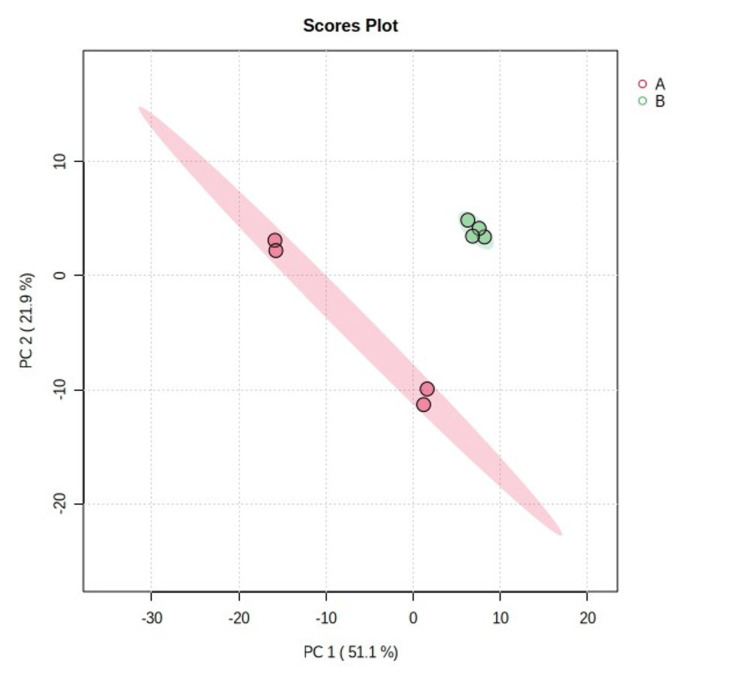
Principal component analysis (PCA) plot showing the distribution of samples or genes in a dataset, focusing on two principal components

The PCA plot obtained in the study shows the distribution of samples or genes in a dataset, focusing on two principal components: PC-1 accounts for 51.1% of the total variation, capturing main patterns, while PC-2 explains 12.1%. The plot helps identify grouping or clustering patterns, as distant samples contribute the most to the observed variation. The percentage values indicate the variability each component explains, allowing researchers to identify relationships or groupings among samples or genes (Figure 3).

**Figure 4:**
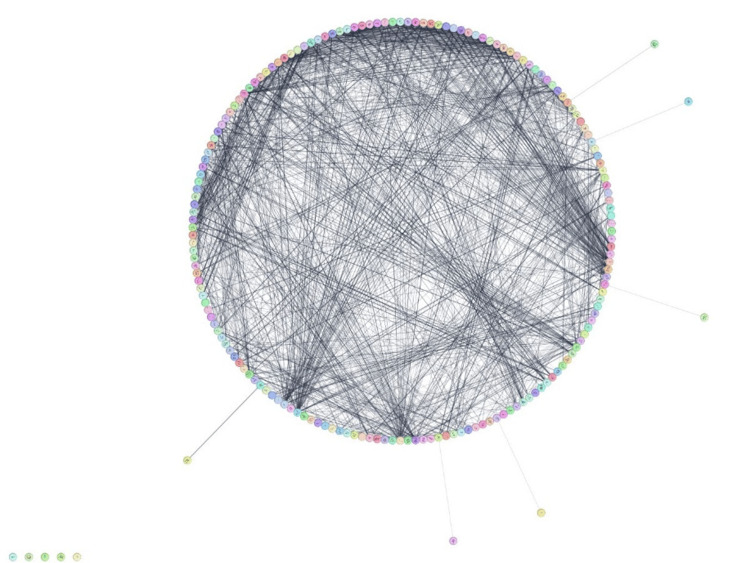
Interactome of differential gene expression in oral cancer

The present study also provides an insightful examination of the protein interactome associated with the differential gene expression hubs. Furthermore, a meticulous analysis was conducted using M code written in Power Query language to identify smaller subsets of cohesive subgroups within larger clusters. Notably, applying CytoHubba analysis successfully identified a distinct sub-cluster among the differentially expressed genes in the hub and top hub genes, namely, SERPINC1, TTR, HSPB1, HP, and APOA1 (Figure 4).

The results of the extra tree classifier model for oral cancer classification indicate high performance in terms of accurately predicting the disease status (Table 1). The AUC value of 0.98 suggests that the model has a high discriminatory power in distinguishing between positive and negative cases of oral cancer. A higher AUC value indicates better model performance, with a value of 1.0 representing a perfect classifier. The CA of 0.97 indicates that the model correctly classified 97% of the instances in the test dataset. This high accuracy suggests that the model effectively differentiates between oral and non-cancer cases. The F1 score of 0.97 demonstrates the balance between precision and recall, with a higher score suggesting better performance. This indicates that the model can accurately classify both positive and negative cases of oral cancer correctly. The precision value of 0.94 shows that when the model predicts a sample as positive (oral cancer), it is correct approximately 94% of the time. This metric indicates the model's accuracy in correctly identifying true positive samples and minimizing false positive predictions. Overall, the results suggest that the extra tree classifier model exhibits high accuracy, precision, and F1 score, indicating its effectiveness in predicting the interactomic hub genes of oral cancer. It demonstrates strong potential for clinical application in guiding decision-making and personalized treatment strategies.

**Table 1:** Extra tree classifier model performance with an accuracy score of 98%

Model	AUC	CA	F1	Precision
Extra tree classifier	0.98	0.97	0.97	0.94

**Figure 5:**
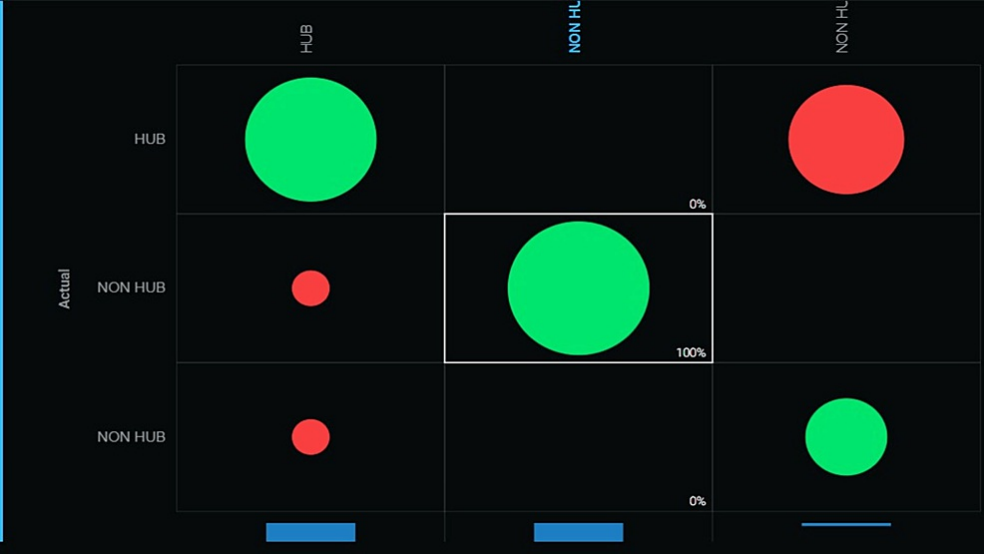
Confusion matrix of true positives and true negatives of nearly 100%

The confusion matrix obtained from the study classifies the model's performance into four categories: true positive (TP), true negative (TN), false positive (FP), and false negative (FN). A true positive rate of nearly 100% indicates that the model correctly identified most positive cases, while a true negative rate of nearly 100% indicates that the model accurately classified most negative cases. In conclusion, a nearly 100% true positive and true negative rate in the confusion matrix indicates that the model performs excellently in accurately predicting hub genes with high sensitivity and specificity (Figure 5).

## Discussion

Saliva, a potential alternative to blood serum and urine for health monitoring, is gaining attention due to its affordability, safety, simplicity, and non-invasiveness. Saliva analysis offers several advantages, including non-invasive sample collection, easy storage, active patient participation, cost-effectiveness, and reduced risk of infection. "Salivaomics" involves the study of various aspects of saliva, such as its genomes, transcriptomics, proteomics, metabolomics, microbiomics, and microRNA. Using techniques like mass spectrometry and proteomics, researchers have identified over 3000 distinct proteins in saliva [11,13-15]. This wealth of data holds promises for customizing therapeutic approaches in the future. The diagnosis of oral cancer relies on comprehensive oral examinations and tissue biopsies for accurate detection. The progression of oral carcinogenesis involves various histopathologic stages and molecular changes, underscoring the importance of proteomics in understanding alterations in gene expression. In specific studies [16,17], researchers analyzed hub genes from saliva samples of individuals with oral cancer and compared them to those from healthy individuals. Identifying these hub genes provides important insights into the molecular pathways involved in oral cancer and can potentially improve diagnostic accuracy and treatment strategies (Figures 1-4).

SERPINC1, a serine protease inhibitor, regulates blood clotting by inhibiting coagulation factors. Mutations in the SERPINC1 gene can lead to inherited thrombophilia. TTR, a carrier protein, binds to thyroid hormones and vitamin A and prevents amyloid aggregation. Mutations in the TTR gene can cause amyloidosis. HSPB1, a small heat shock protein, aids in protein folding and cell cycle regulation. HP, a glycoprotein, binds hemoglobin to prevent oxidative damage and iron loss. Genetic variations in HP can influence susceptibility to certain diseases. APOA1, a major high-density lipoprotein (HDL) component, plays a crucial role in cholesterol metabolism. Mutations in the APOA1 gene can lead to disorders like familial hypoalphalipoproteinemia [1,18,19].

The study found that HSPB1 [16,17] expression in oral squamous carcinoma cells is weak compared to normal human oral keratinocytes, linked to promoter hypermethylation. Treatment with RG108 could induce HSPB1 expression, as seen in primary oral squamous carcinomas. High levels of HspB1 expression in cancer cells contribute to resistance and aggressiveness, leading to poor clinical outcomes in various cancers. HSPB1 is involved in tumor invasion, the formation of metastatic colonies, and resistance to cancer treatments. It targets client proteins to promote resistance to cell death and malignant behavior. Elevated levels of HSPB1 have been found in cancer stem cells, which play a role in their maintenance and induce long-term dormancy. HSPB1 also acts as a sensor of genetic imbalances and enhances cancer cells' resistance to various anti-cancer drugs. Multiplex network analysis identified 46 hub genes for oral cancer, including PIK3CG, PIK3R5, MYH7, CDC20, and CCL4, with high prediction accuracy. These genes may improve understanding of tumorigenesis and molecular events, opening new research routes for multi-omics biological data analysis [20].

One recent study identified 46 hub genes with 96% prediction accuracy, particularly PIK3CG, PIK3R5, MYH7, CDC20, and CCL4, which have significant biological implications for oral cancer and offered new research routes [21]. A study on head and neck squamous cell carcinoma patients identified 65 concordant genes, including a 13-gene panel. Validation in the Oncomine database revealed significant over-expression of all 13 genes, with six genes (CXCL8, CXCL10, FN1, PLAU, SERPINE1, and SPP1) significantly associated with prognosis. This panel could improve diagnostic, prognostic, and treatment approaches [22]. Similar to these previous study results, the current study has identified HSPB1 as one of the interactome hub genes.

The predictive extra tree classifier has shown high accuracy in analyzing interactomic hub genes in oral cancer, with a 98% accuracy rate (Figure 5). This suggests that the identified genes are crucial in oral cancer development and progression.

Limitations

However, the model needs validation in larger, more diverse cohorts to ensure generalizability. In addition, the study may have a potential bias due to prioritizing genes with higher connectivity. However, these prioritizing or hub genes could be better identified by additional in-silico computational approaches.

Future directions include integrating other omics data, developing non-invasive saliva collection methods, and standardizing saliva collection and analysis protocols. Further research is needed to fully understand the potential of interactomic hub genes in oral cancer diagnosis and treatment.

## Conclusions

The predictive extra tree classifier's high accuracy in analyzing interactomic hub genes in oral cancer suggests its potential for improving diagnosis and treatment strategies. However, validation in larger cohorts and integration of omics data are needed for generalizability and robustness. Future research should focus on non-invasive saliva collection methods and larger cohorts.
